# 

**Published:** 1971-10

**Authors:** 


### BRISTOL MEDICO-CHIRURGICAL JOURNAL

OCTOBER 1971

VOL. 86 (iv) No. 320

